# Single-incision laparoscopic colectomy without using special articulating instruments: an initial experience

**DOI:** 10.1186/1477-7819-9-162

**Published:** 2011-12-07

**Authors:** Atthaphorn Trakarnsanga, Thawatchai Akaraviputh, Pakpong Wathanaoran, Chainarong Phalanusitthepha, Asada Methasate, Vitoon Chinswangwattanakul

**Affiliations:** 1 Minimally Invasive Surgery Unit, Division of General Surgery, Department of Surgery, Faculty of Medicine Siriraj Hospital, Mahidol University, Bangkok 10700, Thailand

**Keywords:** Minimally invasive surgery, Single-incision laparoscopic colectomy, Laparoscopic colectomy, Colorectal neoplasms

## Abstract

**Background:**

Single-incision laparoscopic colectomy (SILC) was introduced as a novel minimally invasive technique. The benefits of this technique include reducing number of the incision and cosmetic improvement. Unlike the conventional laparoscopic colectomy, majority of previously reported SILC need to be performed using special curved or articulated instruments. The purpose of this study is to demonstrate our initial experience of SILC, which could be performed using the standard laparoscopic instruments.

**Material and methods:**

Retrospective review of 14 patients who underwent SILC at Siriraj Hospital from May to December 2010, patient's demographic data, perioperative outcomes, early postoperative complications and pathological data were collected and analyzed.

**Results:**

The mean age of all patients was 60 years. The most common operation with SILC was sigmoidectomy (n = 9), followed by right hemicolectomy (n = 2), left hemicolectomy (n = 1), anterior resection (n = 1), and total colectomy (n = 1). The trocar insertion techniques were multi-fascial incision using regular port (n = 11) and GelPOINT^® ^(n = 3). The mean operative time was 155 minutes (range 90-280) and the mean estimate blood loss was 32.1 mL (range 10-100). All patients were successfully operated without conversion. The mean length of hospital stay was 9 days (range 5-20). There was no mortality. The pathological results revealed colorectal cancer (n = 12), neoplastic polyp (n = 1) and Familial adenomatous polyposis (FAP) (n = 1). The mean number of lymph nodes retrieval was 16.6 (range 3-34).

**Conclusion:**

SILC can successfully and safely be performed with standard laparoscopic instruments. This technique might be an alternative procedure to conventional laparoscopic colectomy with better cosmetic result.

## Background

Recently, minimally invasive surgery for colon cancer treatment became more popular. This approach provided better short-term advantages, such as less postoperative pain, rapid return of bowel function, shorter hospitalization and rapidly returns to normal activity, compare to conventional open method. However, there are no differences in terms of post-operative complications and mortality rate compare to conventional open method [1-4]. Moreover, the long-term oncological outcome is not significantly different between both methods [5-8].

In 1992, Pelosi et al. [9] firstly reported the successful technique of single-incision laparoscopic surgery (SILS) which minimizes the number of port from multiple to only one operative port. In the beginning, this technique was used for simple procedures, such as cholecystectomy, appendectomy and nephrectomy [10-12]. Subsequently, SILS was applied to more complex operations, for example, sleeve gastrectomy and colectomy [13,14]. The benefits of SILS include reduce number of the incisions, less postoperative pain [11], improve cosmetic outcome [10,15] and decrease incidence of incisional hernia [14].

After Leroy et al. [16] reported the feasibility of Single-incision laparoscopic colectomy (SILC) in experimental model in 2008, several case series of SILC were widely published. However, SILS has several disadvantages such as the handling of both straight instruments in parallel with the laparoscope through a small incision decreases the freedom of motion for the surgeon and complicates the holding of the laparoscope for the assistant [15]. As a result of limitation of triangulation, majority of reports used special articulating instruments to overcome this problem [17-19]. In this study, we present our initial experiences of SILC successfully perform by using standard non-articulating laparoscopic instruments.

## Material and methods

After the Institutional Review Board approved this study, retrospective review from our prospective maintained database from May to December 2010 was performed. Fourteen SILC procedures were identified, all of these operations performed at Minimally Invasive Surgery Unit, Division of General Surgery, Department of Surgery, Faculty of Medicine Siriraj Hospital. Age, gender, body mass index (BMI), operative time (OPT), estimated blood loss (EBL), early post-operative complications, length of hospital stay (LHS) and pathological data were collected and analyzed.

### Surgical Technique

**Figure 1:**
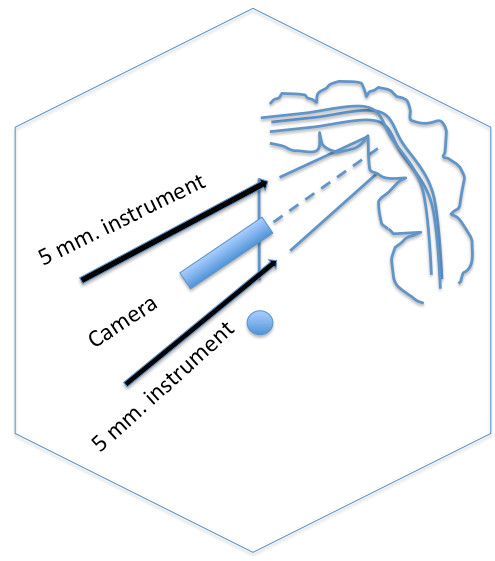
**Diagram showing the port position of three trocars**. The diagram showed the port position of three trocars inserted through a single longitudinal incision using multifascial technique for left side colonic lesion.

**Figure 2:**
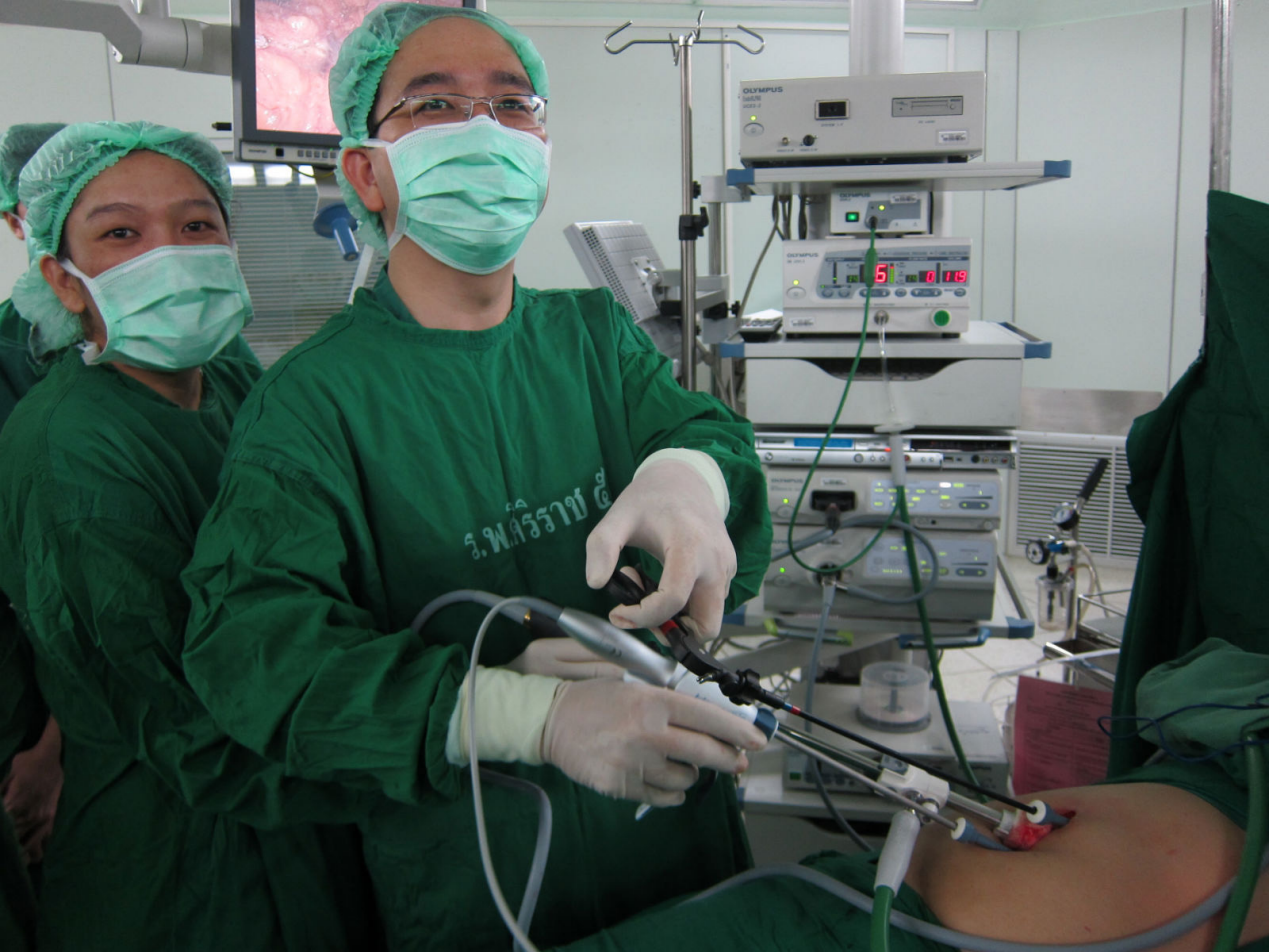
**Picture demonstrating the position of the surgeon**. The picture demonstrated the position of the surgeon and an assistant on right side of the patient performing SILC left half colectomy.

We used two trocar insertion techniques; multi-facial incision (n = 11) and GelPOINT^® ^(n = 3, Applied Medical, Rancho Santa Margarita, CA). For multi-fascial incision, we started with making a small 4-5 cm midline incision. Division of skin and subcutaneous tissue were made except anterior abdominal fascia. Pnuemoperitoneum was created with closed technique by puncturing with the Veress needle awaiting adequate pressure at 15 mmHg. A 12-mm. trocar was introduced to abdominal cavity for a 10-mm., 30-degree camera (Endoeye™, Olympus comp., Tokyo, Japan). Two regular 5-mm. ports were placed at the upper and lower end of incision (Figure 1). In all procedures, three standard non-articulating instruments were used (one bowel grasper, one endohook or vessel sealing device and another one for endoscope) (Figure 2). Median to lateral approach was applied for all operations. The main artery was dissected, double clipped and transected. After the pathological segment of colon was completely mobilized, the pneumoperitoneum was removed. Completion incise of abdominal wall sheath was performed. A small wound retractor (ALEXIS wound retractor S, Applied Medical, Santa Margarita, CA, USA) was applied for wound protection. The specimen was extracted through the midline incision. Colonic resection and making anastomosis were performed extra-peritoneal cavity by double staples or hand-sew technique.

## Results

Fourteen patients were included to this study. The mean age was 60 years (range 23-90). Seven patients were female. The mean BMI was 25.2 kg/cm^2 ^(range 17.3-36.1). Nine patients underwent SILC for sigmoidectomy, two for right hemicolectomy and each one for left hemicolectomy, anterior resection and total colectomy. The mean OPT was 155 minutes (range 90-280) and the mean EBL was 32.1 mL (range 10-100). Extracorporeal anastomosis was created using double staplers (n = 9) or hand-sewn technique (n = 5). There was no conversion in all procedures. The mean LHS was 9 days (range 5-20). There were no postoperative complication and mortality in this study. The pathological results revealed colorectal cancer (n = 12), neoplastic polyp (n = 1) and FAP (n = 1). The mean number of lymph nodes harvested was 16.6 (range 3-34).

## Discussion

After Jacobs [20] firstly reported successfully laparoscopic colectomy in 1990. Varieties of minimally invasive surgical technique for colorectal surgery were introduced, including total laparoscopic colectomy (TLC), Hand-assisted laparoscopic colectomy (HALC), Single incision laparoscopic colectomy (SILC) and Robotic-assisted laparoscopic colectomy (RALC). Each procedure has different benefits as well as limitations. Most surgeons are now convinced of the benefits of the laparoscopic approach in colorectal surgery [1-4]. While advantages of laparoscopic surgery include shorter postoperative hospital stay, early return of bowel function, and decreased complications. Disadvantages of TLC are multiple-port sites in the abdomen and an additional incision for removing the specimen. HALC is suitable in complex procedures and bulky tumor [21]. Moreover, the learning curve is shorter than TLC [21]. For RALC, the most benefit of this approach is total mesorectal excision for rectal cancer. The limitations of RLAC are the restriction of the surgical field and high operative cost.

SILC is the new emerging technique in the past two years. The initial applications of SILS in gastrointestinal surgery were appendectomy and cholecystectomy. Currently, a plenty of reports proposed of safety and feasibility of this technique [14,15,17-19]. In addition, SILC seems to provide improvement in cosmetic result with potential decreased pain by reducing the number of incision[15,22] and possible fewer incidence of post-operative incisional hernia[14]. This technique may generate lower risk of port-side metastasis in malignant cases (only one incision). Disadvantages of SILC are restriction of movement, limitation of triangulation and the axis of camera parallel to the instruments. All of these problems need to be corrected by using the special camera (30 or 45 degree or flexible scope) or the special articulating instruments.

From our series, we started to perform SILC in the selected patients who had an early stage of colon carcinoma. We used two techniques for SILC, one is using GelPOINT^® ^and the other is multi-fascial puncture technique. Most of the cases, we used the later technique because of the simplification of the instrument and lower cost. The limitation of the movement is not great different by the two techniques. We overcome the restriction of the angle by using the 30-degree camera. The dissection was started from medial to lateral approach. One of the reasons using this approach is natural adhesion of the colon to lateral abdominal wall can help us to hold the colon. In addition, familiarity of anatomic landmark same as traditional total laparoscopic approach is another reason.

We used conventional straight laparoscopic instruments such as endohook and bowel grasper. Some cases, we used vessels sealing instruments for soft tissue dissection. Surprisingly, SILC for right half colectomy and sigmoidectomy with conventional straight instruments were not a difficult procedure. Varieties of the laparoscopic procedures can be performed successfully without conversion and with minimal intraoperative blood loss. The operative time is acceptable for the learning period. The lymph nodes were adequately retrieved. One patient needs long hospital stay because of his underlying disease without any operative complications.

## Conclusion

In conclusion, SILC can be successfully and safely performed using standard laparoscopic instruments. When compare to standard laparoscopic colectomy, the potential advantages of the SILS include reduction in incision, decrease postoperative pain, and improve cosmetics. This technique can be done as an alternative method to conventional laparoscopic approach with comparable outcomes.

## Competing interests

The authors declare that they have no competing interests.

## Authors' contributions

AT, TA and VC were the surgeon who performed the SILC operation. TA and AT originated the idea and drafted up the manuscript. AM, PW and CP participated in the operation. TA and PW collected and analyzed the data. TA critically reviewed and edited the manuscript. All authors read and approved the final manuscript.
